# Supplementary material for: Synthesis and Characterization of New Ruthenium (II) Complexes of Stoichiometry [Ru(p-Cymene)Cl2L] and Their Cytotoxicity against HeLa-Type Cancer Cells
Source: Molecules. 2022 Oct 26;27(21):7264. doi: 10.3390/molecules27217264 (PMC9654852; doi:10.3390/molecules27217264)
Supplement: Supplementary file 1 [file molecules-27-07264-s001.zip › molecules-1933539-supplementary.pdf]

Supporting Information

Synthesis and characterization of New Ruthenium (II) Complexes of Stoichiometry [Ru(*p*-cymene)Cl_2L] and their cytotoxicity against HeLa-type Cancer Cells

Marta G. Fuster [1], Imane Moulefera [1], Mercedes G. Montalbán [1], José Pérez [2], Gloria Víllora [1] and Gabriel García [3,*]

[1] Departamento de Ingeniería Química, Facultad de Química, Campus Regional de Excelencia "Campus Mare Nostrum", Universidad de Murcia, 30071 Murcia, Spain

[2] Departamento de Ingeniería Química y Medioambiental, ETSII, Universidad Politécnica de Cartagena, 30203 Cartagena, Spain

[3] Departamento de Química Inorgánica, Facultad de Química, Campus Regional de Excelencia "Campus Mare Nostrum", Universidad de Murcia, 30071 Murcia, Spain

* Correspondence: ggarcia@um.es

NMR spectra

2-ABN

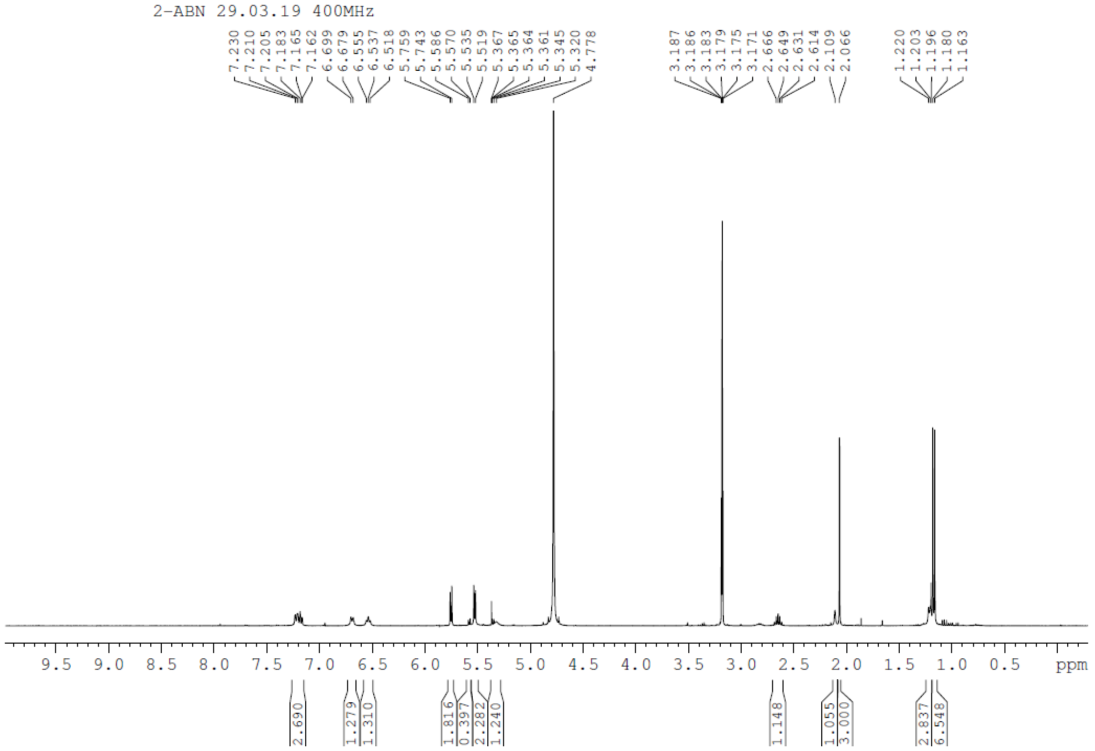

2-ABN.ampl1

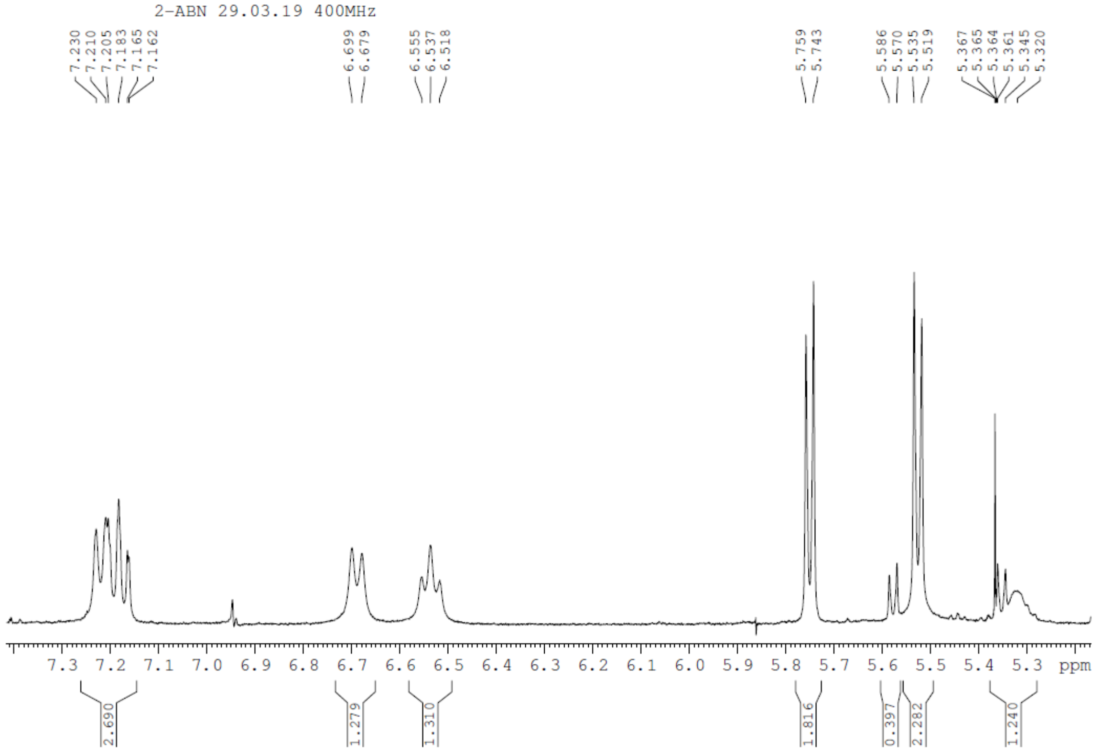

2-ABN.ampl2

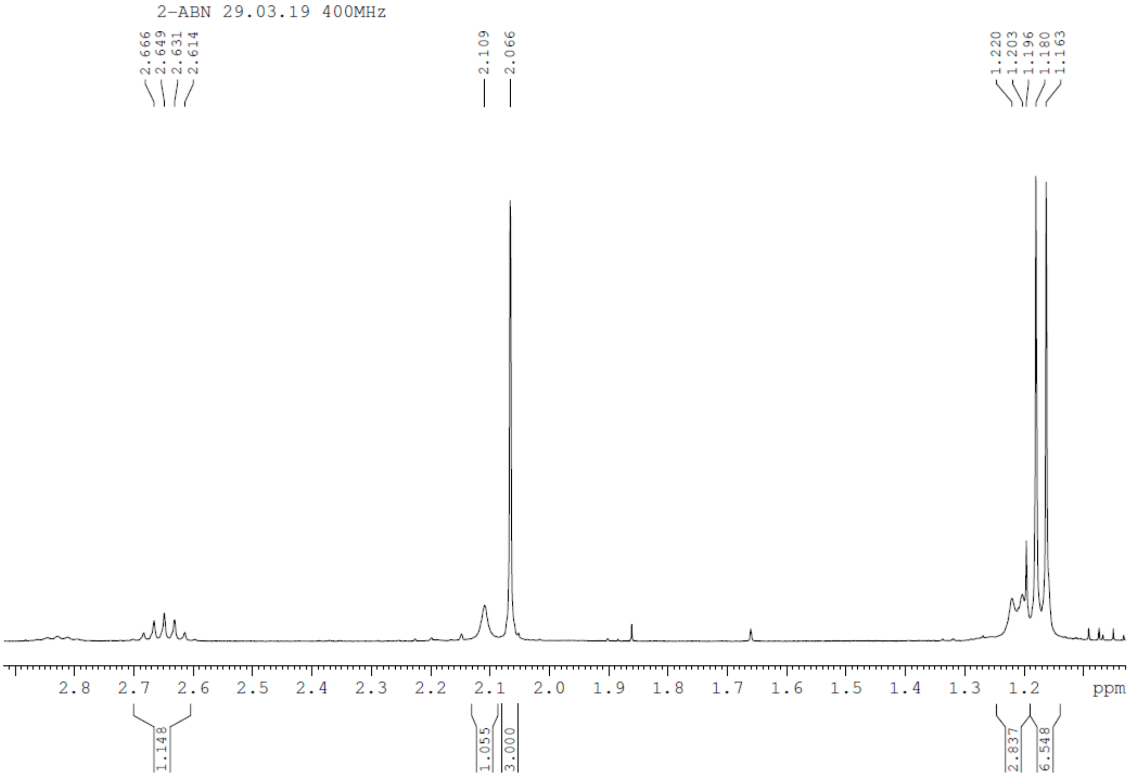

4-ABN 25ºC

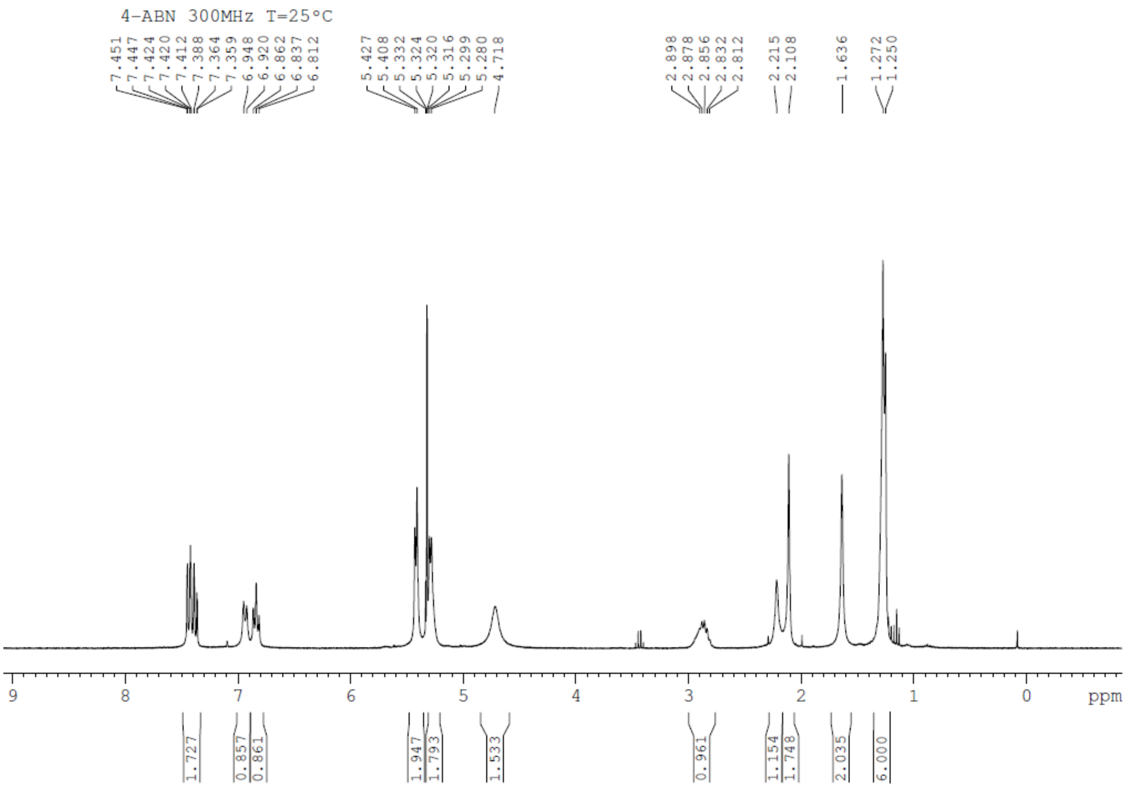

4-ABN 25ºC.ampl1

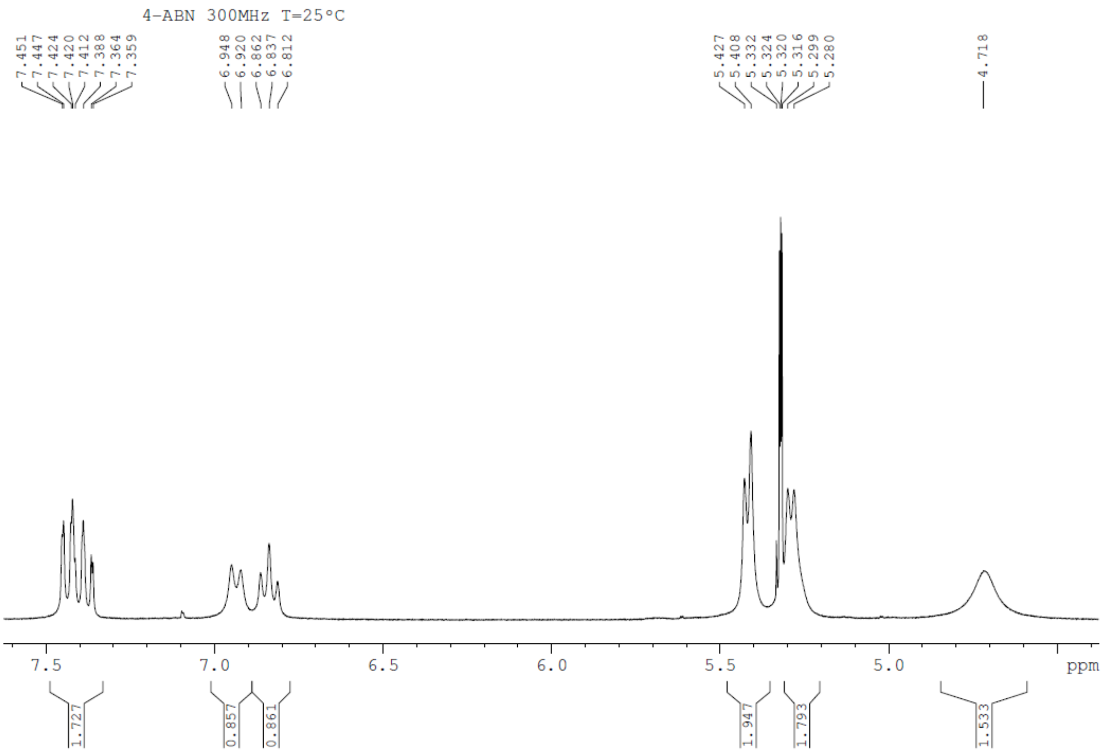

2-AMPYR 1H

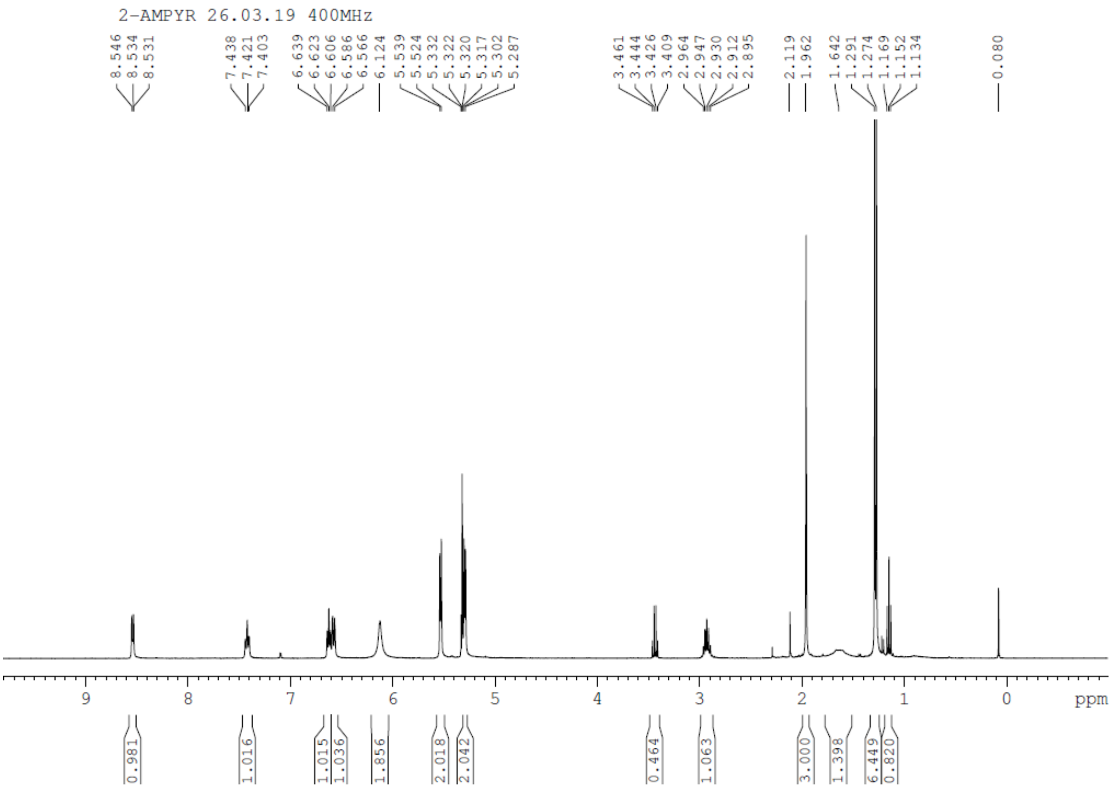

2-AMPYR 1H.ampl1

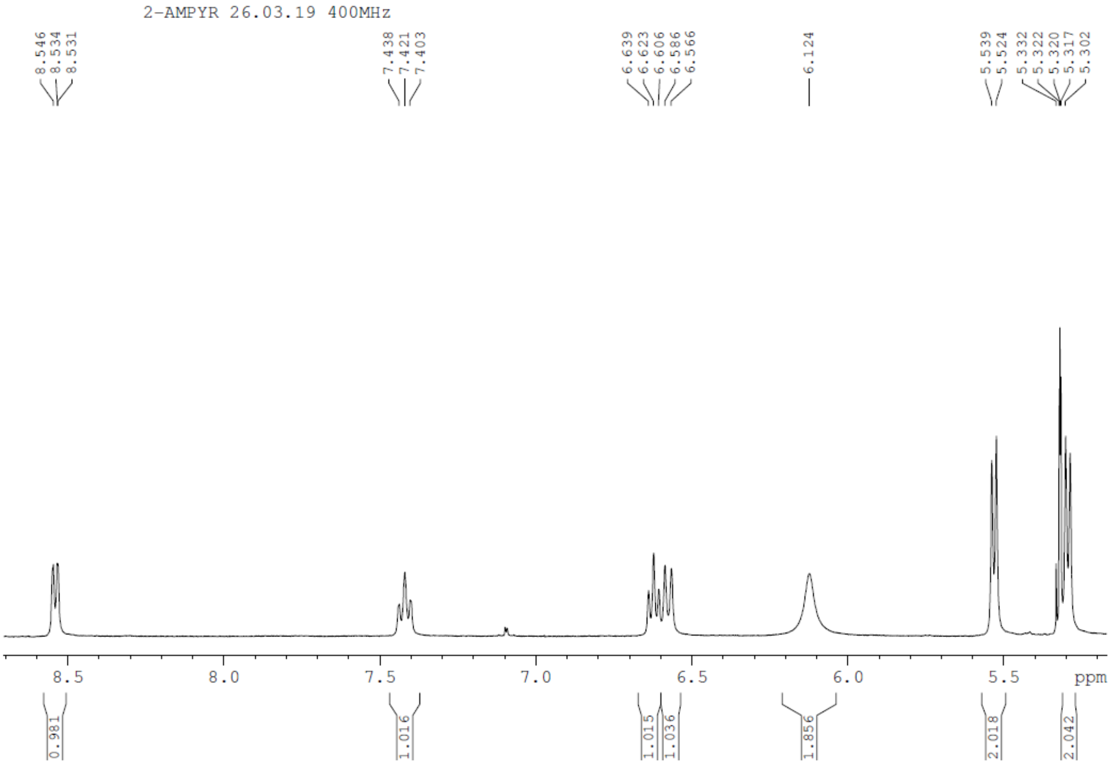

2-AMPYR 1H.ampl2

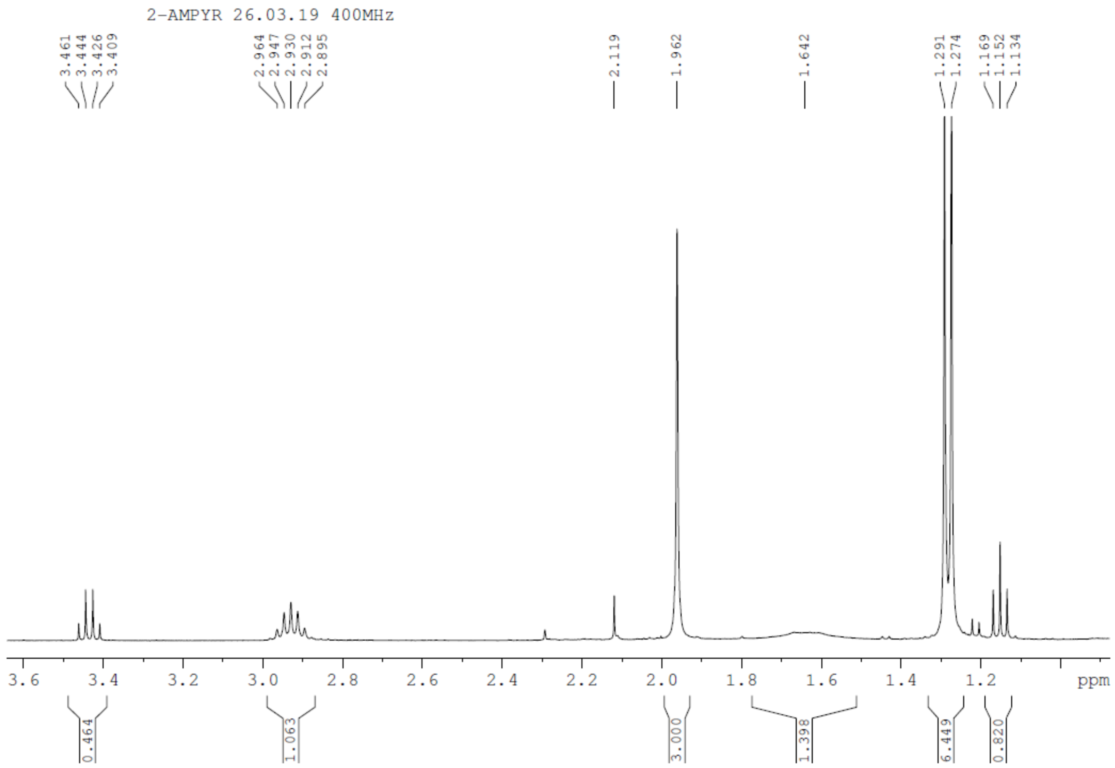

4-AMPYR(PF6) 1H

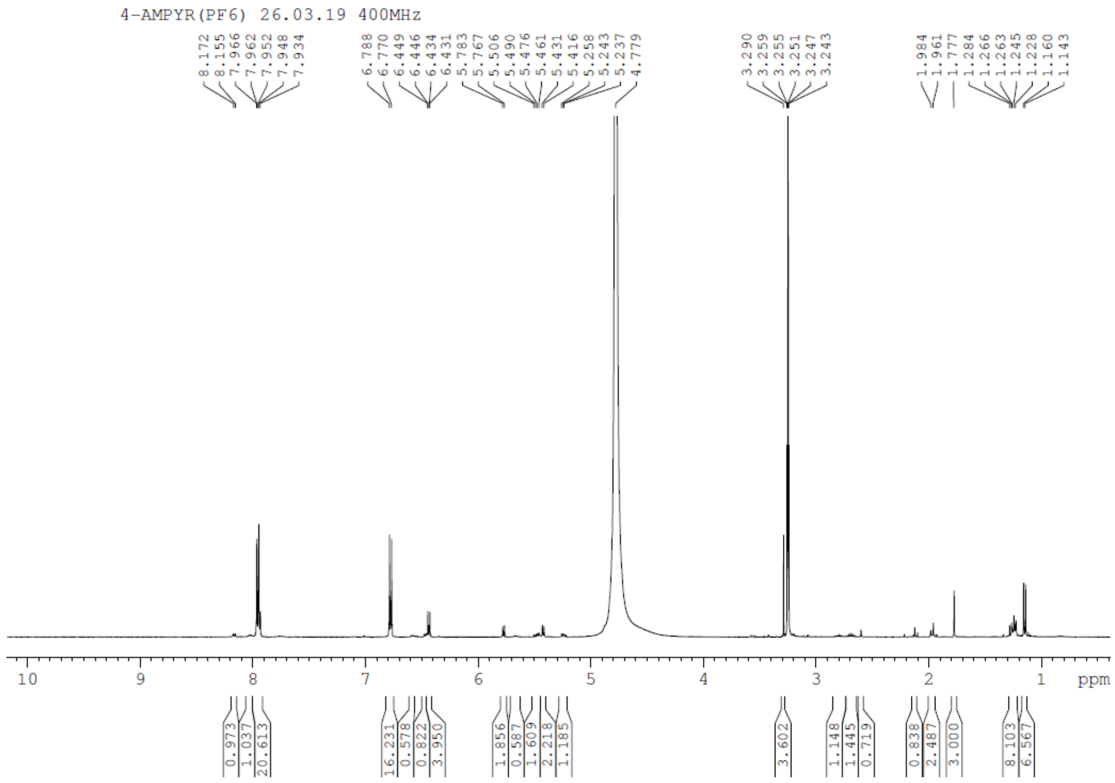

4-AMPYR(PF6).ampl1 1H

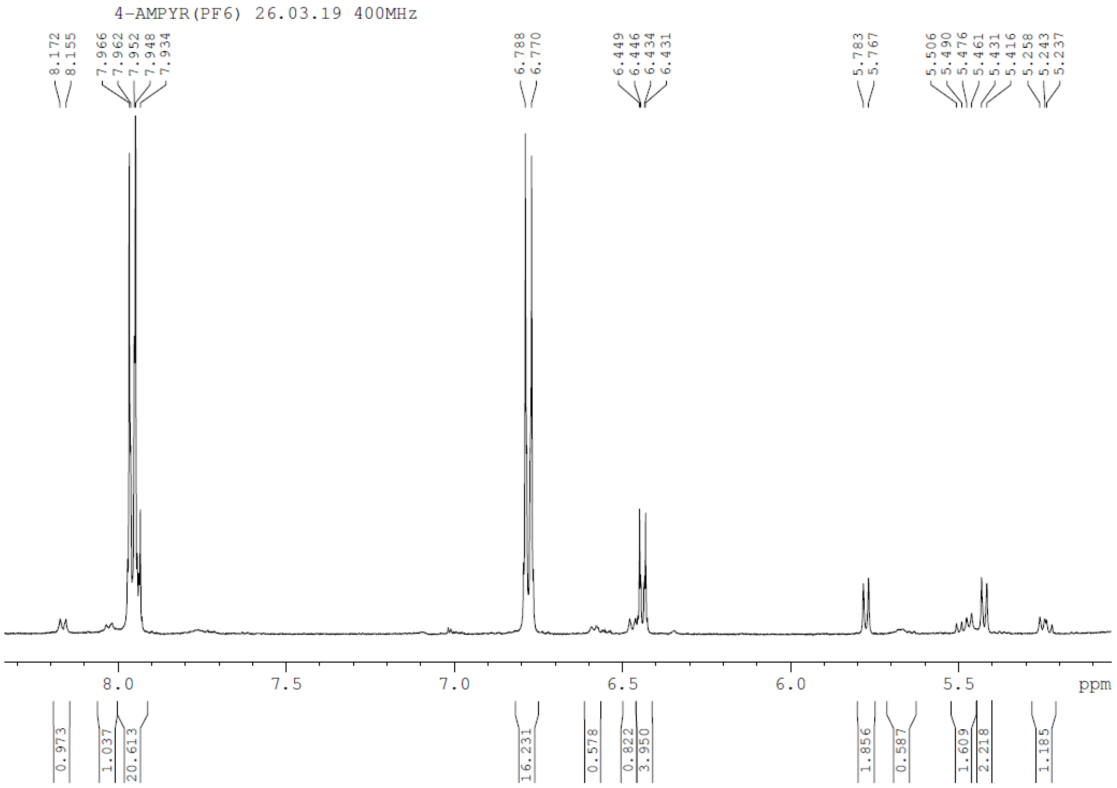

4-AMPYR(PF6).ampl2 1H

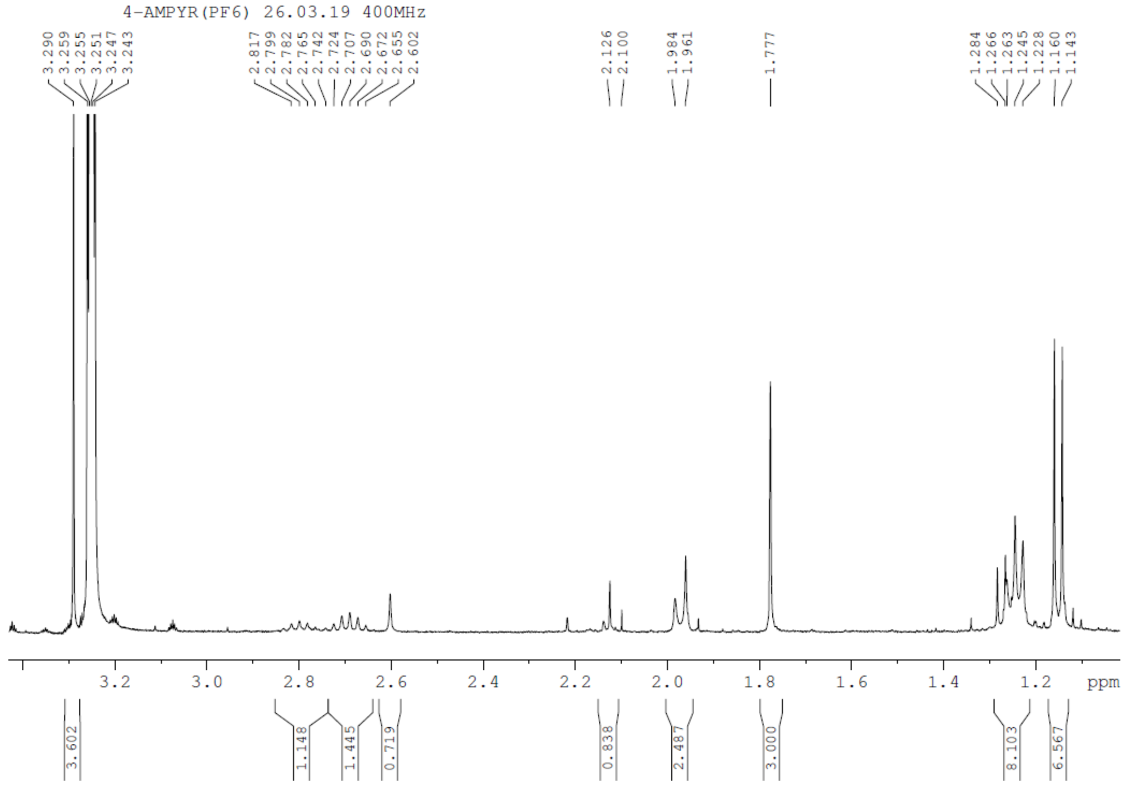

Hirshfeld Surface analysis for Complexe II

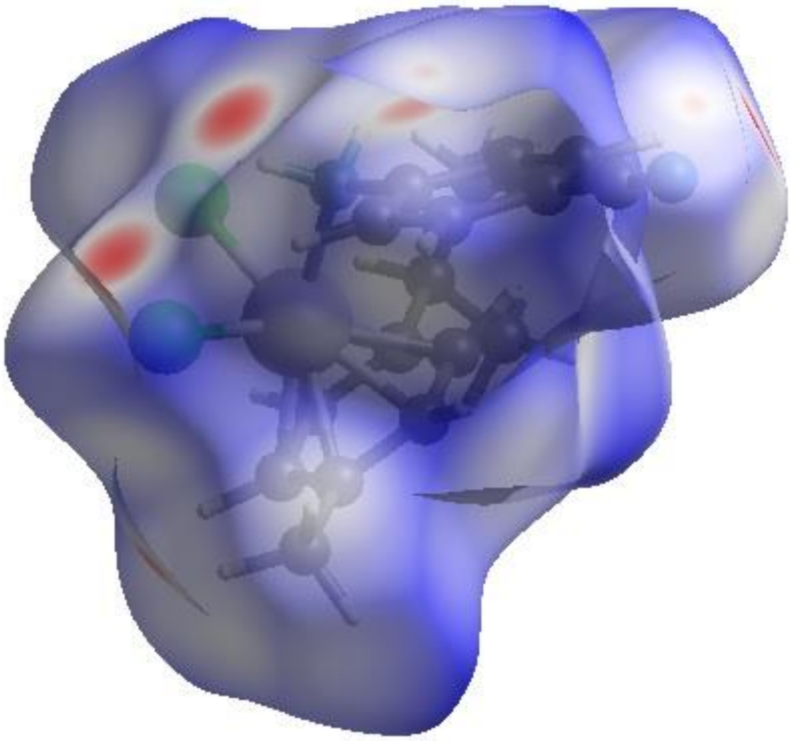

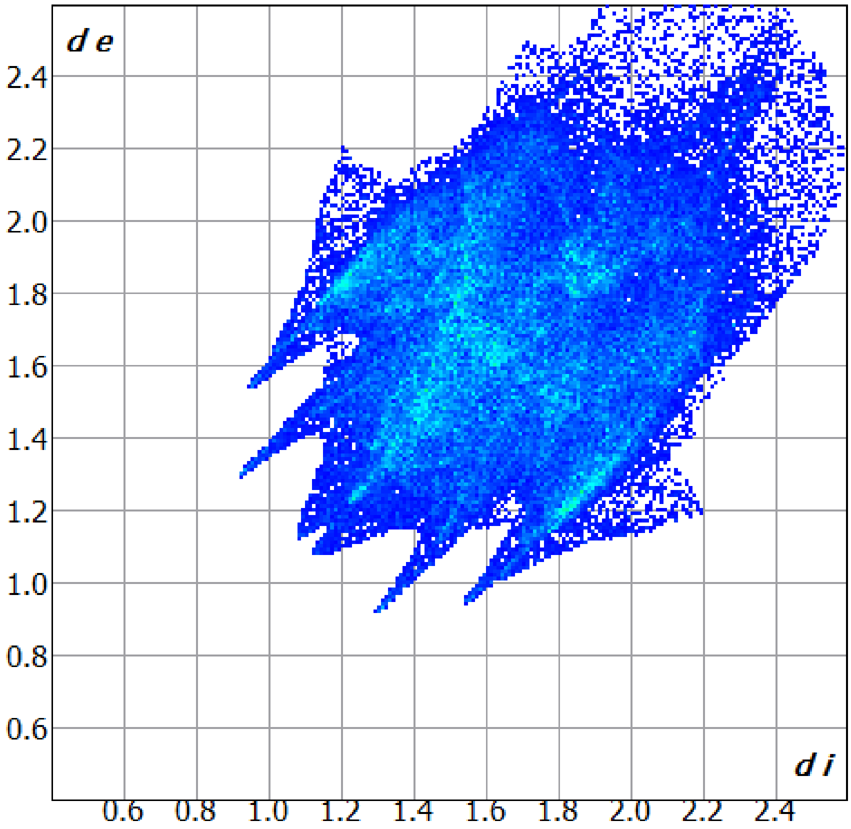

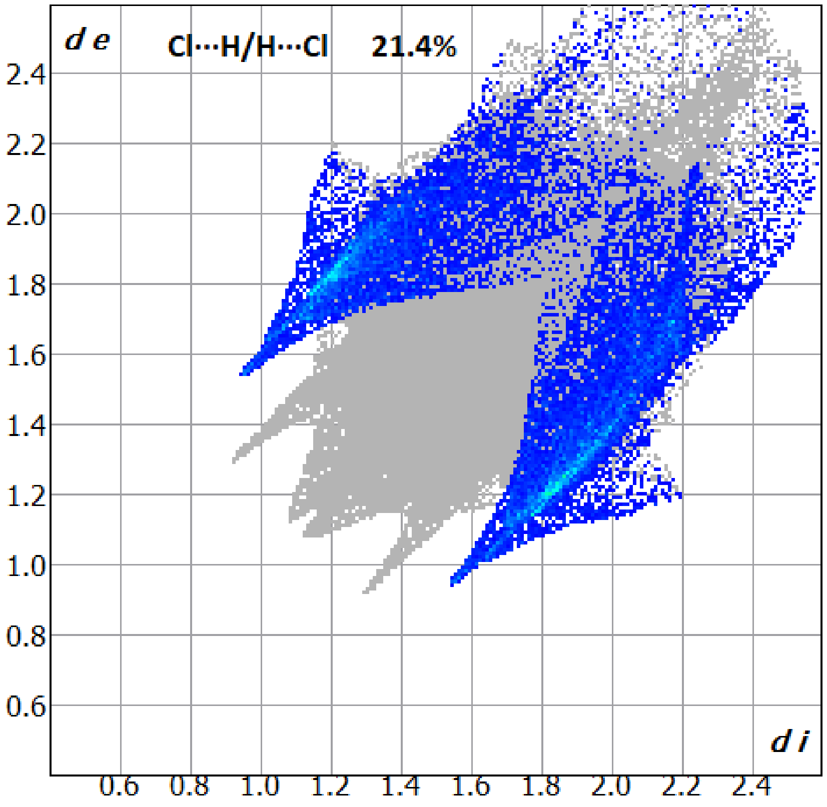

d e
Cl···H/H···Cl
21.4%
2.4
2.2
2.0
1.8
1.6
1.4
1.2
1.0
0.8
0.6
d i
0.6
0.8
1.0
1.2
1.4
1.6
1.8
2.0
2.2
2.4

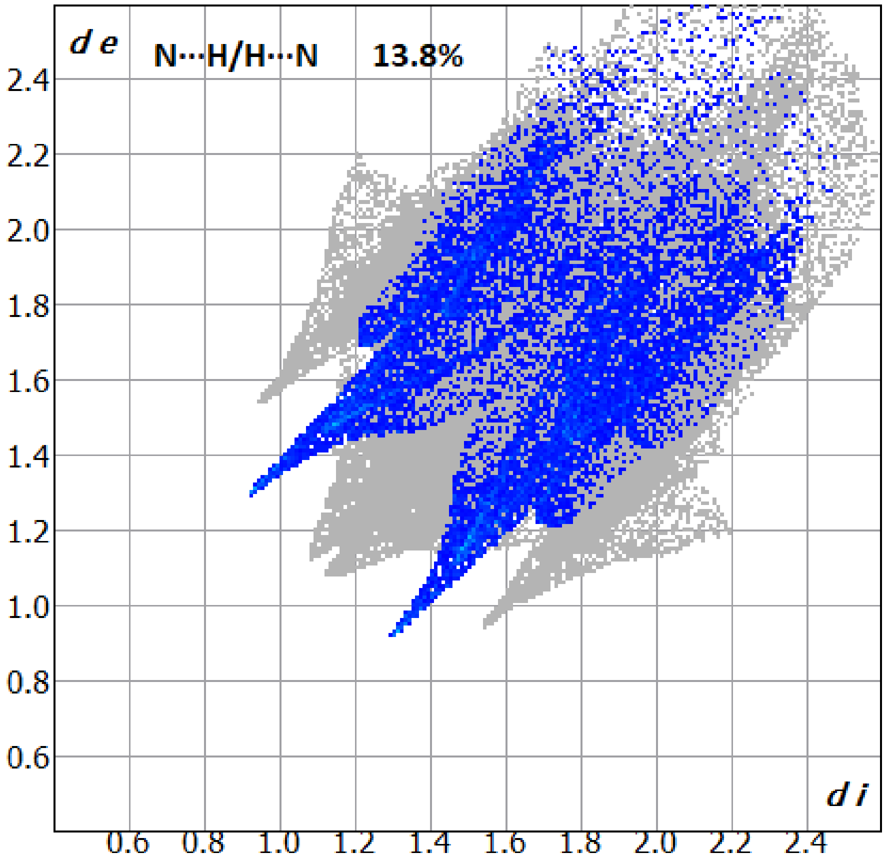

d e
N···H/H···N
13.8%
2.4
2.2
2.0
1.8
1.6
1.4
1.2
1.0
0.8
0.6
d i
0.6
0.8
1.0
1.2
1.4
1.6
1.8
2.0
2.2
2.4

Hirshfeld Surface analysis for Complexe III

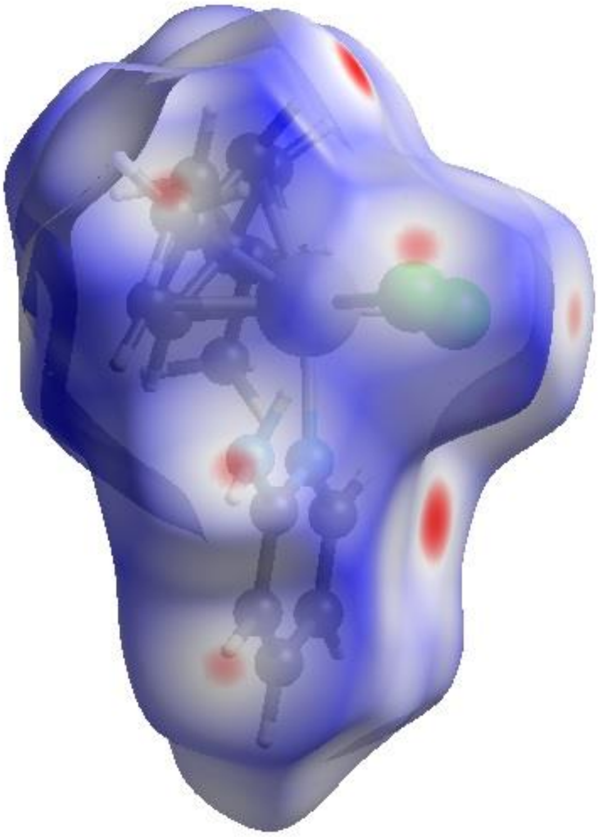

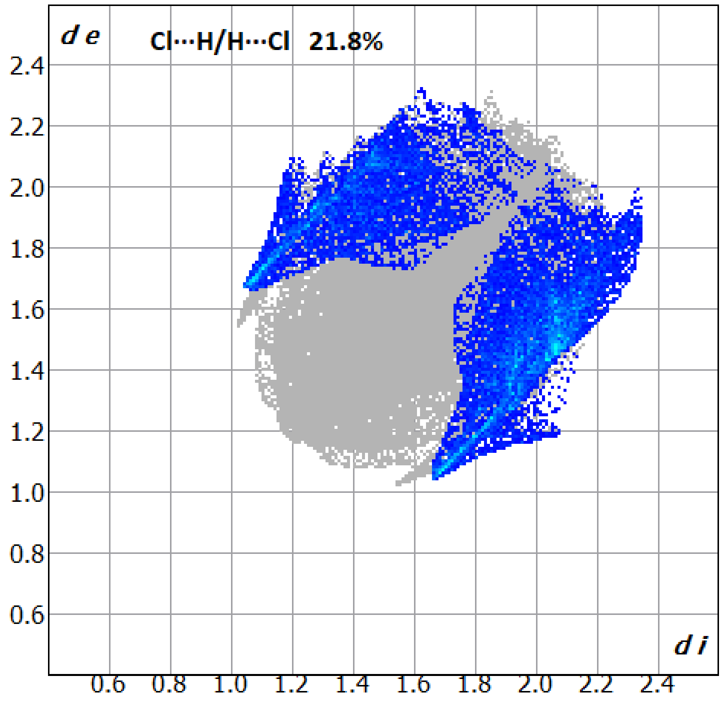

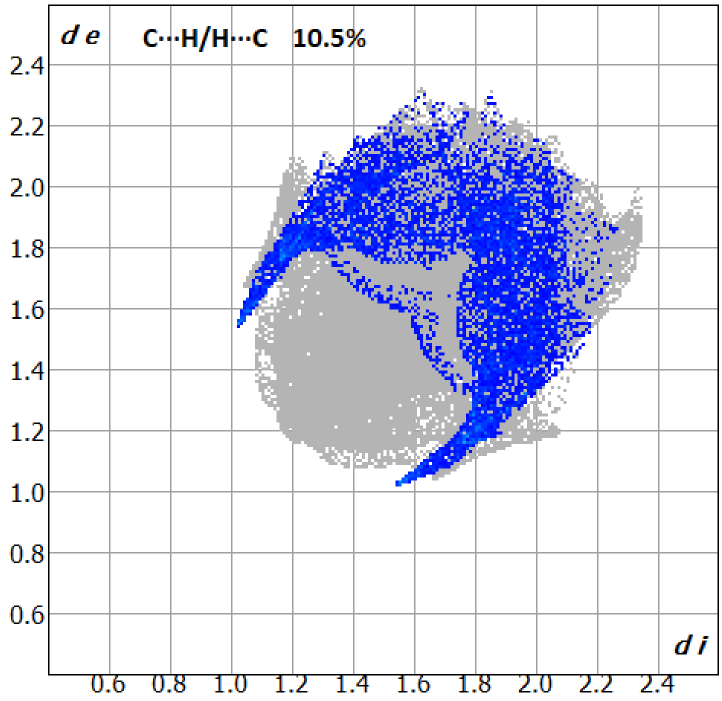
d e
C···H/H···C 10.5%
2.4
2.2
2.0
1.8
1.6
1.4
1.2
1.0
0.8
0.6
d i
0.6 0.8 1.0 1.2 1.4 1.6 1.8 2.0 2.2 2.4

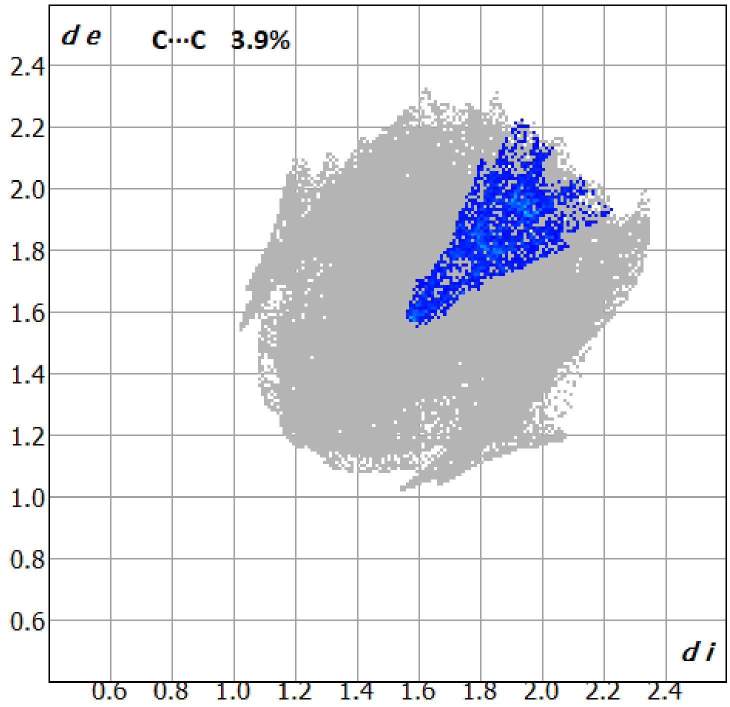
d e
C···C 3.9%
2.4
2.2
2.0
1.8
1.6
1.4
1.2
1.0
0.8
0.6
d i
0.6 0.8 1.0 1.2 1.4 1.6 1.8 2.0 2.2 2.4
